# Supplementary material for: HIVprotI: an integrated web based platform for prediction and design of HIV proteins inhibitors
Source: J Cheminform. 2018 Mar 9;10:12. doi: 10.1186/s13321-018-0266-y (PMC5845081; doi:10.1186/s13321-018-0266-y)
Supplement: Supplementary file 2 — Additional file 2. Source code of HIVProtI web server. [file 13321_2018_266_MOESM2_ESM.zip › HIVprotI_Source-code/hivprotI/weka-3-6-6/doc/weka/gui/beans/EventConstraints.html]

EventConstraints


---


|  |  |  |
| --- | --- | --- |
| **Overview** \| **Package** \| **Class** \| **Tree** \| **Deprecated** \| **Index** \| **Help** \| **Weka's home** | |  |
| **PREV CLASS**   **NEXT CLASS** | **FRAMES**    **NO FRAMES**     **All Classes** |  |
| SUMMARY: NESTED \| FIELD \| CONSTR \| METHOD | DETAIL: FIELD \| CONSTR \| METHOD |  |


---


## weka.gui.beans Interface EventConstraints

**All Known Implementing Classes:**: Associator, ClassAssigner, Classifier, ClassifierPerformanceEvaluator, ClassValuePicker, Clusterer, ClustererPerformanceEvaluator, CrossValidationFoldMaker, Filter, IncrementalClassifierEvaluator, InstanceStreamToBatchMaker, Loader, MetaBean, PredictionAppender, TestSetMaker, TextViewer, TrainingSetMaker, TrainTestSplitMaker

---

``` public interface EventConstraints ```

Interface for objects that want to be able to specify at any given
time whether their current configuration allows a particular event
to be generated.

**Version:**
:   $Revision: 1.3 $

**Author:**
:   Mark Hall

---

| **Method Summary** | |
| --- | --- |
| `boolean` | `eventGeneratable(java.lang.String eventName)`             Returns true if, at the current time, the named event could be generated. |

**Method Detail**

### eventGeneratable

```
boolean eventGeneratable(java.lang.String eventName)
```

:   Returns true if, at the current time, the named event could be
    generated.

    :   **Parameters:**: `eventName` - the name of the event in question **Returns:**: true if the named event could be generated


---


|  |  |  |
| --- | --- | --- |
| **Overview** \| **Package** \| **Class** \| **Tree** \| **Deprecated** \| **Index** \| **Help** \| **Weka's home** | |  |
| **PREV CLASS**   **NEXT CLASS** | **FRAMES**    **NO FRAMES**     **All Classes** |  |
| SUMMARY: NESTED \| FIELD \| CONSTR \| METHOD | DETAIL: FIELD \| CONSTR \| METHOD |  |


---
